# The Promise of Blood-Based Biomarkers in Early Detection and Care of Alzheimer’s Disease

**DOI:** 10.3126/nje.v16i2.98106

**Published:** 2026-08-03

**Authors:** Muneeb Khawar, Pugazhandhi Bakthavatchalam

**Affiliations:** 1 King Edward Medical University, Lahore, Pakistan; 2 Associate Professor of Anatomy, American University of Antigua

Alzheimer's disease (AD) is one of the most important health concerns among the older adults. More than 55 million people are currently living with dementia worldwide and the majority of these have AD. By 2050, this figure is estimated to increase almost three times [1]. Previously, the diagnosis of AD required the use of invasive procedures such as lumbar puncture to examine CSF, or expensive PET scans, both of which are uncomfortable for patients. There is, however, a considerable shift towards the use of blood-based biomarkers (BBMs). These are simple blood tests that can be done at an earlier stage. They are able to identify proteins like phosphorylated tau and amyloid-beta that predict AD before any symptoms are present [2]. Other bodily fluids and tissues are also being analyzed such as saliva and tears for presence of other notably alpha-synuclein. Recent approvals of targeted therapies such as lecanemab are making BBMs increasingly important in the diagnosis of AD. The health system, researchers and policy makers must promote their adoption in routine use to make a real difference [1].

## Biological and Pathological Perspectives

BBMs are closely linked with the classic indicators of AD, including amyloid plaques and tau tangles, which are both available by taking blood sample [3]. p-tau217 and p-tau181 are markers of protein folding and accumulation problems, and the Aβ42/Aβ40 ratio is related to amyloid-related problems. Neurofilament light is a marker for nerve damage and glial fibrillary acidic protein is a marker for brain inflammation [4]. High levels of p-tau217 correlate strongly with AD. Combining p-tau217 with NfL or GFAP increases predictive accuracy, with an area under the curve of approximately 79% for any dementia and 71% for AD-specific cases over a decade. Remarkably, the negative predictive values of these tests are greater than 90% [4]. Immune dysfunction, disturbances in neuronal connections and vascular injury are underlying problems involved in AD. The emerging biomarkers, including p-tau205 and mid-region tau fragments, can offer insight into disease staging and progression, which is of great value in monitoring treatment efficacy [3].

## Diagnostic Accuracy and Clinical Applications

BBMs are effective in identifying AD risk long before mild cognitive impairment occurs. For instance, plasma p-tau217 is closely aligned with positive tau PET scans [2]. This correlation enables physicians to exclude AD pathology confidently, reducing the reliance on invasive procedures. In general populations, elevated levels of p-tau217 and NfL correspond to a 3- to 4-fold increase in dementia risk among individuals in the top quartiles [4]. Current guidelines recommend using BBMs to stratify patients in specialized settings, with sensitivities of at least 90% and specificities of 75% or higher. They may also serve as substitutes for PET or CSF tests when necessary, both of which show over 90% efficacy [1]. However, the effectiveness of BBMs can differ among various demographic groups, especially among diverse ethnic backgrounds where testing has been insufficient [5]. Therefore, combining these tests with comprehensive clinical evaluations is essential to minimize the risk of misdiagnosis.

## Patient and Caregiver Impacts: Psychological Dimensions

Receiving an AD diagnosis is challenging, particularly when it is made late, leading to increased anxiety, depression, and caregiver burnout. BBMs have the potential to transform this experience by initiating discussions about care plans and support earlier, thus alleviating uncertainty. Research suggests that communicating negative results with predictive values above 95% can relieve anxiety for individuals [4]. However, in the absence of confirmatory tests, these negative results may generate additional concern, particularly for asymptomatic individuals, where the evidence for screening remains limited. Early detection, conversely, opens avenues for innovative treatments such as lecanemab [6], which can substantially enhance day-to-day living. Nonetheless, disparities in access affect non-white communities disproportionately, contributing to heightened emotional stress from increased misdiagnosis rates [5]. It is estimated that approximately 7.2 million adults over 65 years in the U.S. have AD, with many more undiagnosed. Improving education is essential to address stigma and foster resilience within these communities [5].

## Societal and Economic Implications

BBMs have the potential to provide significant benefits for society, despite some challenges that need to be addressed. In the United States, the cost of Alzheimer’s disease (AD) exceeds $780 billion annually when considering all factors [4]. Implementing BBMs could accelerate diagnosis, support workforce participation, and reduce costs through early intervention. Globally, among higher-risk populations, AD prevalence ranges from 10% to 30% [4]. BBMs could help create equity by being integrated into standard medical appointments. However, coverage limitations by insurance companies can hinder progress [5]. Additionally, in regions lacking specialists, patients may face wait times of over a year [5]. Establishing a strong connection between BBMs and established diagnostic methods, such as amyloid PET scans, could advocate for broader insurance coverage and lessen the financial strain on patients receiving treatment. Moreover, innovative solutions like dried blood spot sampling could facilitate remote testing, thus expanding accessibility [3].

## Conclusion

BBMs play a crucial role in combating Alzheimer’s disease and should be prioritized. To embed them in routine primary care, it is essential for governments and health organizations to finance evaluations in varied demographics, establish coverage policies, and integrate BBMs into existing workflows. Their accuracy, comparable to traditional invasive methods, enables early detection of AD, allowing for lifestyle adjustments and therapies that may hinder or delay disease progression. For patients, this means gaining a sense of control, meeting their needs, and moving forward without the burden of an uncertain diagnosis. This is not merely a transient trend; embracing BBMs could fundamentally alter the perception of aging in a future where Alzheimer’s disease diminishes in prominence.

## References

[1] Tahami Monfared AA Phan NN Pearson I. A systematic review of clinical practice guidelines for Alzheimer's disease and strategies for future advancements. Neurology and therapy. 2023;12(4):1257-84. https://doi.org/10.1007/s40120-023-00504-6 PMid:37261607 PMCid:PMC10310649 10.1007/s40120-023-00504-6 37261607 PMC10310649

[2] Chong JR Ashton NJ Karikari TK. Blood-based high sensitivity measurements of beta-amyloid and phosphorylated tau as biomarkers of Alzheimer's disease: a focused review on recent advances. Journal of Neurology, Neurosurgery & Psychiatry. 2021;92(11):1231-41. https://doi.org/10.1136/jnnp-2021-327370 PMid:34510001 10.1136/jnnp-2021-327370 34510001

[3] Lofstrom MA. Blood-Based Biomarkers and the Future of Alzheimer's Disease Diagnostics [online] n.d. [cited 2025 Sep 2]. Available from: URL:https://media.beckmancoulter.com/en/blog/diagnostics/blood-biomarkers-for-alzheimers-screening

[4] Deverka PA Arias JJ Lin GA Zwerling J Phillips KA. Alzheimer's disease blood-based biomarker testing: A stakeholder-informed assessment of coverage considerations. Journal of Alzheimer's Disease. 2025;105(2):433-42. https://doi.org/10.1177/13872877251329394 PMid:40170393 PMCid:PMC12143641 10.1177/13872877251329394 40170393 PMC12143641

[5] Hunter TR Santos LE Tovar-Moll F De Felice FG. Alzheimer's disease biomarkers and their current use in clinical research and practice. Molecular Psychiatry. 2025;30(1):272-84. https://doi.org/10.1038/s41380-024-02709-z PMid:39232196 10.1038/s41380-024-02709-z 39232196

